# Editorial Comment: Adjuvant Single-Dose Upper Urinary Tract Instillation of Mitomycin C After Therapeutic Ureteroscopy for Upper Tract Urothelial Carcinoma: A Single-Centre Prospective Non-Randomized Trial

**DOI:** 10.1590/S1677-5538.IBJU.2021.01.05

**Published:** 2020-11-18

**Authors:** João Paulo Martins de Carvalho

**Affiliations:** 1 Hospital Federal Cardoso Fontes Serviço de Urologia Rio de JaneiroRJ Brasil Serviço de Urologia, Hospital Federal Cardoso Fontes, Rio de Janeiro, RJ, Brasil

Gallioli A ^1^ ^2^, Boissier R ^1^ ^3^, Territo A ^1^, Vila Reyes H ^1^, Sanguedolce F ^1^, Gaya JM ^1^, et al.

## COMMENT

This article aims to demonstrate the efficacy of mitomycin C infusion right after the ureteroscopy procedure for ablation of urothelial carcinomas in patients submitted to kidney preservation protocol.

Fifty-two patients were enrolled from April 1, 2015 to August 31, 2018, submitted to laser ablation followed by a single dose of mitomycin- (ASDM).

The study was approved by the ethics committee. All patients with urothelial lesion < T2 and those in which endoscopic resection seemed to be feasible were included. Patients with bladder lesions greater than 3.0 cm or incomplete ablation of the upper urinary tract were excluded. The control group was composed of patients with mitomycin C intolerance but with similar radiological aspects.

The technique was described as intravenous antibiotic prophylactic therapy with second-generation cephalosporin, 30 minutes before surgery. Initial cystoscopy and trans urethral bladder resection (TUR-B) after ureteroscopy if necessary. Semi rigid ureteroscope was used for tumors of the distal third of the ureter and flexible ureteroscope with the use of an access sheath when ablation in the pelvis, renal calyx, proximal and middle third of the ureter. The ablation was performed with a laser fiber with a holmium or thulium generator depending on which was available and subsequent drainage of the renal pelvis with double or single J stent.

The immediate infusion of mitomycin C at the dosage of 40mg/40 mL diluted in 100 ml saline solution, was made up do six hours postoperatively, through the ureter catheter or intra-vesicaly when with single or double J stent. Catheters were removed up to 14 days postoperatively.

The primary objective was to evaluate the safety and efficacy of ASDM. The secondary, evaluate recurrence. Follow-up was based upon computed tomography scans every 03 months or ureteroscopy every 06 months, for two years.

The patients were then grouped into two groups. ASDM- A, and control- B.

The mean urothelial tract carcinoma (UTUC) size was 15.1 mm. UTUCs were in the calyx, pélvis and ureter in 15 (29%), 19 (37%), and 25 (49%) cases, respectively; multifocality was present in 33%. For postoperative drainage, a single-J stent was used in 19 cases (76%) and a double-J stent in six (24%), comprising four patients in whom UTUC was located in the ureter and two in whom a grade I/II ureteral injury was observed after removal of the ureteral sheath.

The use of mitomycin C did not promote significant side effects, observing only one urinary retention by clots in a patient with single kidney and hematuria in two patients.

In the ASDM group, two patients (8%) were assigned to nephroureterectomy for high-grade or recurrent neoplasia. The overall survival rate was 90.6% (39/43), and the cancer-specific survival rate was 97.6% (42/43). Eight patients (18.6%) had maintenance treatment consisting in weekly upper tract instillations (mitomycin in five and BCG in three). The oncological outcomes of ASDM were evaluated by comparing 17 ASDM patients (group A) with the 18 patients who did not receive any other adjuvant treatment after therapeutic ureteroscopy (group B).

Median follow-up was 18 months. Urothelial recurrence occurred in 23.5% of patients in group A vs 55.5% in group B. In groups A and B respectively, urothelial recurrence consisted in upper tract recurrence in 17.6% (3/17) vs 33.3% (6/18) and bladder recurrence in 21.4% (3/14) vs 31.3% (5/16).

Bladder and local recurrence were metachronous in two patients (11.8%) of group A and synchronous in one patient (5.5%) of group B.

The risk of urothelial recurrence was significantly higher in patients with high-grade UTUC or previous or concomitant bladder tumor.

## ASDM reduced the risk of recurrence 7.7-fold

The authors conclude that the administration of mitomycin C in a single postoperative dose decreases the chance of recurrence of urothelial carcinoma.

The criticisms of the article cited by the authors themselves mention the small number of individuals in each group, despite a similar demographic distribution and no statistical difference between them, except age and tumors of higher degree in group A.

Paradoxically Baboudjian et al. obtained a considerable large number of bladder recurrences even with the systematization of diagnostic ureteroscopy including using ureteral access sheaths (1).

It can be assumed that when manipulating the upper urinary tract for ablation of a urothelial carcinoma, mitomycin can be infused in order to decrease its recurrence.

Only as historical documentation, it is worth mentioning that one of the most cited articles in kidney preservation for urothelial carcinomas, used BCG through percutaneous access, in those patients not candidates for nephroureterectomy.

Of a total of 37 patients with 41 treated kidney units, there was a 38% progression with subsequent death and 33% were still alive after a 42 months follow-up (2).

Only 18 years separates, these publications and serve to document the advance of minimally invasive therapeutic options.

This article has the merit of documenting the systematization of renal ablation therapy, as well as the infusion of topical Mmitomycin C with favorable short-term results, including decreased intra vesical recurrence.
